# MicroRNAs in Tissue Regeneration: Lessons from Animal Models

**DOI:** 10.3390/ijms262010043

**Published:** 2025-10-15

**Authors:** Sarah E. Walker, Alicia Piazza, Robert L. Carlone, Gaynor E. Spencer

**Affiliations:** Department Biological Sciences, Brock University, St. Catharines, ON L2S 3A1, Canada; sarah.ewalker@outlook.com (S.E.W.); ap15rq@brocku.ca (A.P.); rcarlone@brocku.ca (R.L.C.)

**Keywords:** miRNA, mRNA, tissue regeneration, heart, limb, spinal cord

## Abstract

MicroRNAs (miRNAs) are a class of small noncoding RNAs that regulate gene expression. Over the past two decades, multiple studies have established the importance of miRNAs in regulating a variety of biological processes, one of which includes regenerative repair. Although many miRNAs have been shown to regulate the expression of genes that are required for regeneration, few studies have extrapolated these findings from cell culture to in vivo animal models or reported comparative work between regenerating and non-regenerating systems. Here, we review the most current literature highlighting the role of distinct miRNAs in regulating the repair of different tissues, focusing on the heart, limb and spinal cord. In exploring existing work, we emphasize the importance of using animal models to provide foundational knowledge that could potentially lead to future therapeutic strategies to allow for functional regenerative repair in humans.

## 1. Introduction

Regeneration has fascinated scientists for centuries, with some of the earliest reports of regeneration provided in the 1700s [1,2]. Regeneration is widespread throughout the animal kingdom but is highly variable between different species [3]. While some invertebrates such as planaria [4] and acoels [5] are capable of whole-body regeneration, vertebrates are more restricted in their regenerative capabilities. Indeed, only a few vertebrate species, such as fish and amphibians, exhibit tissue-specific regenerative capabilities, including appendage, organ, or nervous system regeneration [6,7]. Meanwhile, humans and most mammals appear to have largely lost the ability to regenerate after extensive injuries.

The few vertebrates that are capable of regenerative repair serve as exciting model systems to investigate the basis of regeneration and to understand its limitations in mammals. Comparative work on regeneration-competent vertebrates with non-regenerating mammals has thus enhanced our understanding of the basic molecular and cellular events that underlie successful regeneration.

Through the exploration of the molecular signaling pathways that promote regeneration, microRNAs (miRNAs) have emerged as well-established regulators of tissue repair [8,9,10,11,12,13]. These small, noncoding RNAs are approximately 22 nucleotides in length and bind to complementary mRNA sequences, whereby they ultimately prevent the mRNA from being translated into a functional protein [14]. Such post-transcriptional methods of modifying gene expression enable the precise control of many different proteins that promote key events during regenerative repair, including cellular dedifferentiation [8], proliferation [8,10,15,16,17] and differentiation [13].

**Figure 1:**
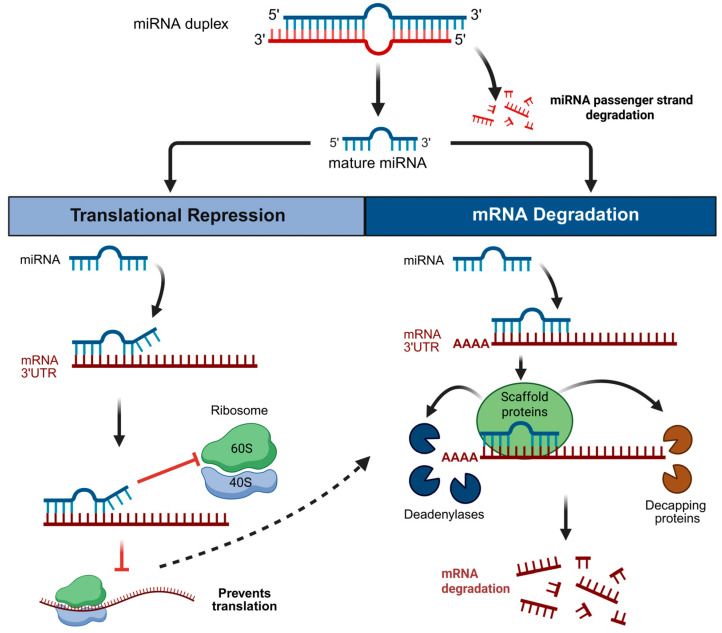
Conventional microRNA mode of action. Mature microRNA binding with complementary base pairing to the 3′ untranslated region (UTR) of target mRNAs interferes with ribosome binding and translation initiation, thus resulting in translational repression (red lines indicate inhibition). The dotted line indicates that translational repression can (but not always) be followed by mRNA degradation. If proceeding to degradation, there is recruitment of scaffolding proteins (Trinucleotide Repeat containing (TNRC) 6A/B/C in mammals, GW182 in flies, AIN-1/2 in nematodes) [41,42], which in turn recruit deadenylases and decapping proteins, leading to endonuclease-mediated degradation of the mRNA strand. Created with BioRender.com.

The regulation of miRNA biogenesis and the functional implications for miRNA-mRNA target interactions have previously been reviewed [18,19,20]. Summarizing briefly, miRNA biogenesis produces three RNA species: primary (pri-), precursor (pre-) and mature miRNA [14]. The mature miRNA consists of a 5p strand (arising from the 5′ arm of the pre-miRNA hairpin) and a 3p strand (arising from the 3′ arm) [19]. One strand, known as the guide or mature strand (Figure 1, blue) is loaded into an Argonaute protein to form a functional silencing complex, whereas the other strand (Figure 1, red), also known as the passenger strand, is often degraded. However, both 5p and 3p strands have the potential to serve as guides, and in some cases mature miRNA-3p and -5p arms are both functional, although this is often tissue/cell type and/or context-dependent [21,22,23].

Canonical miRNA-mRNA binding involves strong complementary base-pairing between a small seed region on the 5′ end of the miRNA with the 3′-untranslated region (3′UTR) of an mRNA. In the conventional model, complementary binding results in translational repression followed by mRNA deadenylation and ultimately degradation of the mRNA strand [24,25,26] (Figure 1). While the mechanisms that direct whether a mRNA strand will undergo translational repression or degradation are not yet completely understood, this decision is likely determined by the complementarity of the miRNA to the mRNA [27,28]. Deviations from this conventional model can, however, occur; translational repression and deadenylation of mRNA targets can occur independently or in parallel to facilitate both mRNA repression and decay [29,30]. For example, in zebrafish embryos (pre-gastrulation), miRNAs predominantly inhibit translation without mRNA decay, yet following gastrulation miRNA action follows the conventional model of repression followed by decay [31]. To fully capture the actions of miRNAs in all biological contexts, translation should thus be examined with comprehensive approaches such as targeted mRNA reporter systems, ribosome profiling and proteomics [18,32].

The location of binding sites can also influence miRNA-induced silencing. That is, non-canonical binding sites also exist in the 5′UTR and coding regions of target mRNAs [33,34,35]. The mechanisms and reasons underlying non-canonical miRNA-mRNA binding remain unclear, yet evidence suggests that these sites predominantly lead to translational repression, whereas canonical 3′UTR binding ultimately triggers decay [36]. To further probe these mechanisms, mRNA targets must be accurately predicted, which can be done using in silico prediction tools. Several of these tools are freely available and can predict potential miRNA-mRNA binding sites, but while some focus only on canonical sites [37], others (i.e., mintURLS, miRWalk and DeepMirTar) also include non-canonical sites [35,38,39]. Some tools, such as TargetScan, can even estimate the repression level expected from the number of binding sites [40]. Although these tools are diverse and many cover a diverse population of vertebrate species, they currently lack information on transcripts originating from non-conventional animal models.

Comprising an estimated 1–5% of animal genes, miRNAs are considered one of the most abundant classes of gene regulators [43]. They are also highly conserved across the animal kingdom, showing little to no divergence in their sequences or the mRNAs that they target. For example, approximately 80% of all human miRNAs are also found in the pufferfish [44]. This high degree of conservation indicates that miRNAs may have similar functions across different animal lineages and thus may target the same mRNAs in different species. Indeed, the mature miR-9 sequence is identical in many animals (from the fruit fly to humans) with a similar biological role, ultimately targeting mRNAs regulating neuronal proliferation and differentiation [45]. Due to their high level of conservation, miRNAs serve as key regulatory molecules which could be harnessed as a therapeutic strategy to enhance regenerative repair, simply by altering their expression patterns.

While many miRNAs have been shown to play a role in tissue repair, the cell types involved in the regeneration of different appendages and organs vary drastically. This often makes it difficult to determine whether a single miRNA plays a tissue-specific role in regenerative repair or rather plays a more global response to injury. Here, we review the most recent literature that outlines the role of specific miRNAs in the regeneration of various organs and appendages in animal models. Specifically, we focus on epimorphic regeneration [46], a process involving the regeneration of a replacement organ in situ and requiring cell proliferation, dedifferentiation and/or activation of stem cells. We also identify miRNAs that play a more global role in regulating the cellular events that underlie regenerative repair in multiple organs and/or tissues in different model systems.

## 2. Heart Regeneration

Many regeneration-competent vertebrates such as the newt [47], axolotl [48] and zebrafish [49] are able to successfully repair large portions of their heart after extensive damage or injury. While mammalian species have the ability to generate cardiomyocytes throughout their life, they possess a limited proliferative capacity as adults, thus lacking the ability to synthesize new heart muscle in response to traumatic injury [50,51]. While heart injuries themselves may be less prominent in humans, heart failure and disease are global concerns and remain one of the leading causes of death worldwide [52]. Importantly, both heart injuries and heart failure require the production of new cardiomyocytes to successfully repair damaged heart tissue. Indeed, during a myocardial infarction approximately 25% of the cardiomyocytes found in a single heart ventricle die within a few hours [53]. More extensive heart failures often result in the death of more than 40% of the cardiomyocytes [53]. As such, understanding the underlying mechanisms that regulate pro-regenerative responses after cardiomyocyte damage are of critical importance for human health.

**Figure 2:**
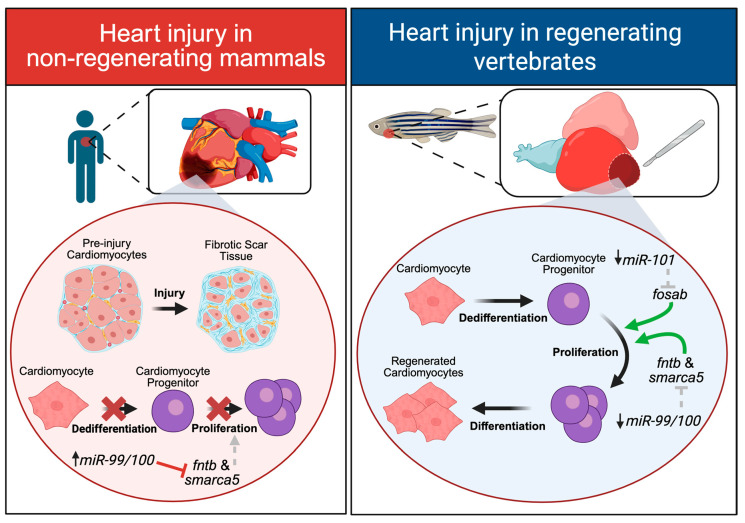
Cell-type responses to heart injury. In non-regenerating mammals, heart injury leads to the formation of fibrotic scar tissue, and surviving cardiomyocytes do not dedifferentiate or proliferate in response to injury. After a heart injury, miR-99-5p/100-5p expression remains high, and it continues to downregulate its target genes, *fntb* (*beta subunit of farnesyl-transferase*) and *smarca5*, (*SWI/SNF-related matrix associated actin-dependent regulator of chromatin subfamily a*) which results in impaired cardiomyocyte proliferation. After a heart injury in regenerating vertebrates like the zebrafish, no fibrotic scar tissue forms, and cardiomyocytes dedifferentiate into progenitor cells that rapidly proliferate to give rise to newly regenerated cardiomyocytes. Both miR-101-3p and miR-99-5p/100-5p are downregulated which results in the subsequent increase in their target genes *fosab* (*FBJ murine osteosarcoma viral oncogene homolog Ab*) and *fntb/smarca5*, respectively, to promote cardiomyocyte proliferation. Green arrows indicate activation. Grey dotted arrows (**left panel**) indicate relief of activation and grey dotted lines (**right panel**) indicate relief of inhibition. Red crosses indicate inhibition. Created with BioRender.com.

Following a heart injury in regeneration-competent animals, mature cardiomyocytes surrounding the injury site dedifferentiate into cardiac progenitor cells, which then rapidly proliferate to replace the damaged heart tissue [54]. Adult mammals, however, do not exhibit cardiomyocyte dedifferentiation or proliferation but instead replace damaged cardiac tissue with fibrotic scar tissue [55]. This noncontractile scar tissue leaves the heart with dramatically impaired cardiac output by altering both its mechanical and electrical properties [55]. Different cellular responses to a traumatic heart injury thus occur in regenerating and non-regenerating animals, resulting in either cardiomyocyte regeneration or scar tissue deposition, respectively (Figure 2).

### microRNAs in Heart Regeneration

Comparing uninjured zebrafish heart tissue to heart tissue harvested 6 h after ventricle injury, microarray profiling revealed a dramatic change in the expression of several miRNAs [10]. Notably, miR-101a-3p was significantly downregulated in the first 3 days after heart injury, then exhibited a dramatic upregulation between 7 and 14 days, before returning to basal levels by 30 days post-injury. Using heat-inducible transgenic zebrafish strains to tightly regulate the expression of miR-101a-3p, researchers found that depleting miR-101a-3p immediately after a heart injury increased the proportion of proliferating cardiomyocytes, while sustained elevation of miR-101a-3p expression reduced cardiomyocyte proliferation [10]. This initial downregulation of miR-101a-3p immediately after injury was necessary to upregulate the expression of its target mRNA, *fosab* (*FBJ murine osteosarcoma viral oncogene homolog Ab*, the zebrafish ortholog of mammalian *fos*), to promote the proliferation of cardiomyocytes necessary for kick-starting regenerative repair. Though *fosab* was the target of interest, this downregulation of miR-101a-3p 3 days post injury also enhanced the expression of other downstream target genes including *cpeb1a* (*cytoplasmic polyadenylation element binding protein 1a*), *stmn1a* (*survival motorneuron 1 gene*; encodes stathmin protein), *mkp1* (*mitogen-activated protein kinase phosphatase-1*), *MYCN* (*v-myc avian myelocytomatosis viral oncogene neuroblastoma derived homolog*) and *cox2* (*cytochrome c oxidase*). When the researchers sustained the downregulation of miR-101a-3p for 14 days after injury (when levels would normally increase), scar tissue, composed of collagen and fibrin, formed at the injury site. The binding of miR-101a to its target mRNA, *fosab*, at 7–14 days post-injury was thus necessary to promote the scarless wound healing normally seen in control animals. This work emphasizes how the precise temporal control of miR-101a-3p expression is necessary to initially promote cardiomyocyte proliferation and later, to prevent the formation of scar tissue [10].

Comparative work between regeneration-competent zebrafish and regeneration-incompetent mice uncovered important differences in the expression of multiple miRNAs after a heart injury. In zebrafish, a heart injury resulted in a dramatic reduction in miR-99-5p/100-5p, leading to the upregulation of its target mRNAs *fntb* (*beta subunit of farnesyl-transferase*) and *smarca5* (*SWI/SNF-related matrix associated actin-dependent regulator of chromatin subfamily a*), which are known to play important roles in cell proliferation [8]. The chemical inhibition of FNT*β* resulted in a reduction in cardiomyocyte proliferation and impaired heart regeneration [8]. Likewise, the use of miR-99-5p/100-5p mimics to upregulate the expression of miR-99-5p/100-5p after a heart injury also resulted in blocking regenerative repair by reducing the number of proliferating cardiomyocytes. In mice, however, miR-99-5p/100-5p expression remained high after a heart injury, resulting in low levels of the target mRNAs, *fntb* and *smarca5*. The use of lenti-viral vectors encoding anti-miRs to inhibit miR-99-5p/100-5p after a heart injury in adult mice increased the number of dedifferentiated and proliferating cardiomyocytes and led to improvements in heart regeneration [8]. Importantly, this inhibition of miR-99/100-5p also led to a significant reduction in fibrotic tissue scarring compared to controls [8]. Despite its differential expression following injury, these findings suggest a conserved function for miR-99-5p/100-5p in regulating cardiomyocyte dedifferentiation and proliferation between regeneration-competent and -incompetent animals. By manipulating the expression patterns of this miRNA, we might thus potentially improve outcomes to promote functional heart regeneration in mammals.

## 3. Limb Regeneration

While regeneration-competent animals such as the axolotl and newt are capable of fully regenerating their limbs after a traumatic injury, mammals have a far more limited capacity for repair. The adolescent mouse is, however, well-known for its ability to regenerate its digit tip [56], and multiple case studies have also reported some capacity for human digit tip regeneration in children [57,58]. However, humans and other mammalian species clearly lack the heightened regenerative capabilities that are demonstrated in other animals and are unable to regenerate their entire limbs after injury or amputation. In 2019, an estimated 550 million people worldwide suffered from limb loss, primarily due to amputations resulting from traumatic injury or diabetes [59]. Diseases that lead to limb loss are growing in prevalence [60], so understanding the basics of limb regeneration may provide knowledge to help prevent disease or aid in trauma recovery.

In regeneration-competent animals, research has focused on understanding the cell-type responses to a limb injury. After a limb amputation, regeneration begins with a rapid wound healing phase. Mature cells then surround the stump tissue and will generally dedifferentiate into multipotent progenitor cells, which rapidly proliferate to form a mass known as the regeneration blastema. Eventually, these undifferentiated cells within the blastema begin to differentiate into various cell types to successfully regenerate the missing limb [61,62]. Elegant tracing studies in the regeneration-capable axolotl discovered that dedifferentiating cells in the limb have a restricted lineage and keep a molecular memory of their tissue origin [63]. For example, the vast majority of dedifferentiating cartilage cells give rise to new cartilage and do not form muscle or epidermis [63]. While both the newt and axolotl can successfully regenerate their limbs after an amputation, there appears to be some species-specific differences in the cells that contribute to the regeneration of muscle. In the newt (*Notophthalmus viridescens*), mature muscle fibers dedifferentiate to ultimately produce newly regenerated muscle tissues [64], whereas in the axolotl, muscle dedifferentiation does not contribute to the regeneration blastema. Instead, satellite cells (a type of muscle stem cell), are recruited from the mature tissue and act as the main contributor to newly regenerated muscle tissue [64].

**Figure 3:**
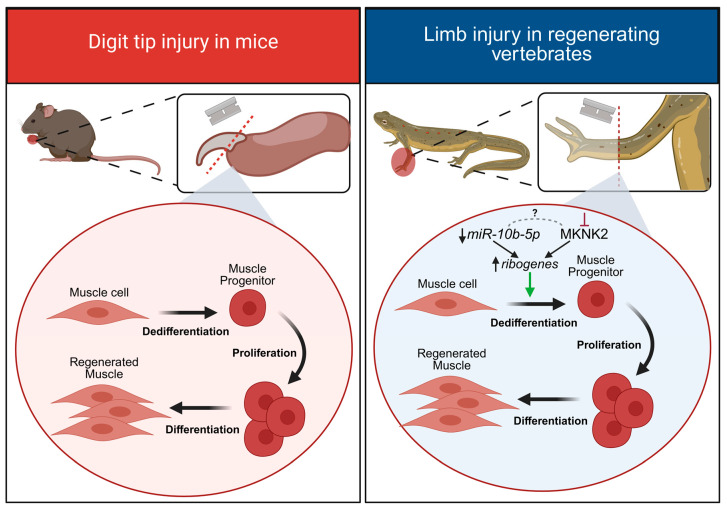
Examples of cell-type responses to a limb injury. Digit tip injury in mice elicits a regenerative response in which muscle cells dedifferentiate into progenitor cells that proliferate to ultimately differentiate into newly regenerated muscle tissue. In regeneration-competent vertebrates like the newt, a more extensive limb amputation also evokes a regenerative response. After injury, muscle cells dedifferentiate into progenitor cells that rapidly proliferate to ultimately dedifferentiate into newly regenerated muscle. The downregulation of miR-10b-5p after injury is important for the upregulation of ribogenes required for protein synthesis; pharmacological inhibition (red line) of MKNK2 (mitogen-activated protein kinase-interacting serine/threonine-protein kinase 2) has the same effect. Though they functionally converge, a molecular interaction between miR-10b-5p and MKNK2 is unclear (grey dotted line). Created with BioRender.com.

In the few instances where mammals are capable of limited limb regeneration, similar cellular events of dedifferentiation and proliferation occur after injury. After digit tip amputation in mice, a regeneration blastema also forms, which similarly comprises a pool of dedifferentiated lineage-restricted progenitor cells [65]. Cre-lox fate mapping demonstrated that dedifferentiated mesodermal tissue exclusively gave rise to new mesoderm tissue, and dedifferentiated ectodermal tissue only gave rise to newly regenerated ectoderm [65]. Collectively, studies using animal model systems have demonstrated that after a limb amputation or digit tip injury, successful regeneration occurs through the recruitment of satellite stem cells and/or the dedifferentiation of mature cell types to give rise to the newly regenerated limb tissue (Figure 3).

### microRNAs in Limb Regeneration

Recent work comparing limb regeneration in two different newt species, *Pleurodeles waltl* and *N. viridescens*, explored the role of miR-10b-5p in directing the dedifferentiation of mature muscle fibers into proliferating muscle progenitors [17]. It was shown that miR-10b-5p was downregulated in skeletal muscle immediately following a limb amputation (Figure 3). This was important to ensure that target ribogenes (*rpS29*, *rpL30*, *rpL4*, *rpL15*, *rpL27A*, encoding ribosomal proteins) were upregulated after limb injury to promote protein synthesis. Though the exact interaction between them remains unclear, both downregulation of miR-10b-5p and inhibition of MKNK2 (mitogen-activated protein kinase-interacting serine/threonine-protein kinase 2) functionally converge to increase protein synthesis and promote cell cycle re-entry; inhibition of MKNK2 alone also produced an increased expression of miR-10b-5p target ribogenes. In later stages of regeneration (late-blastema; when proliferation is decreasing), miR-10b-5p expression returned to pre-injury levels. Interfering with the initial downregulation of miR-10b-5p levels (using a mimic injection) led to a significant decrease in the expression of multiple ribogenes, an increase in MKNK2 immunofluorescence and a reduced number of proliferating cells in the regeneration blastema, thus impairing limb regeneration [17]. Ultimately, this study highlights how miRNAs can tightly regulate the transitional state of cells during regenerative repair; specifically, miR-10b is downregulated in early stages of limb regeneration to promote muscle dedifferentiation and cell proliferation but is later upregulated when cells within the regeneration blastema begin to differentiate into mature cell types [17]. As axolotls do not rely on mature muscle dedifferentiation after a limb injury but instead recruit satellite stem cells, future studies could determine whether miR-10b-5p might also regulate limb regeneration in the axolotl.

During axolotl limb regeneration, miR-21 was shown to be upregulated [66] and was also highly expressed in the regenerating limb of *N. viridescens* [17] (though its role was not further examined). However, examination of the supplementary data revealed that miR-21-5p was more abundant in both the blastema and the regenerating stump (muscle cells) of *N. viridescens*, compared to uninjured tissue. Two variants of miR-21-3p were also identified and though both were abundant in the limb stump, their expression in the blastema varied; miR-21-3p (ACAGCAG) was less abundant, whilst miR-21-3p (AACAACA) was more abundant compared to controls [17]. It is thus possible that miR-21 is important for early limb regeneration across a number of salamander species [17,66].

Indeed, upregulation of miR-21 is important for the early stages of limb regeneration in a different species of newt, *Cynops orientalis* [67]. In this species, miR-21-5p expression was considerably higher than other miRNAs, even at 0 dpa, and was upregulated even further during time points corresponding to wound healing and limb bud formation (1–5 dpa, 5–10 dpa) [67]. Weighted gene co-expression network analysis and gene ontology enrichment analysis identified key target genes; miR-21-5p was predicted to target *Rhou* (*Ras homolog family member U*) which encodes a RhoGTPase, and *Gpd2* (*Glycerol-3-phosphate dehydrogenase 2*) which encodes a mitochondrial enzyme. The authors proposed that downregulation of *Gpd2* could lead to a reduction in mitochondrial activity and may result in a metabolic shift towards glycolysis, promoting regeneration. Additionally, temporal downregulation of *Rhou* would provide the necessary switch required for cells to cease migration and begin differentiation into the appropriate cell types needed to rebuild the lost limb [67]. Thus, the combined reduction in *Rhou* and *Gpd2* expression (as a potential consequence of upregulated miR-21-5p), could promote limb regeneration. Interestingly, *Rhou* can also be targeted by several other miRNAs (miR-210-5p, miR-150-5p, miR-194-5p, miR-141-5p, miR-11260b, miR155-5p) and *Gpd2* can also be targeted by miR-194-5p.

An earlier study in *C. orientalis* examined the temporal regulation of the microRNAome, transcriptome and proteome in parallel during limb regeneration [68]. The number of regeneration stages examined provided the opportunity for a comprehensive analyses of morphological and physiological changes during limb regeneration, including wound healing at 3 dpa (days post-amputation), limb bud formation (7 dpa), blastema cell proliferation (14 dpa), chondrogenesis (development of cartilage) (30 dpa) and digit formation (42 dpa). In this instance, miR-21-3p (rather than -5p) was detected and differentially expressed across the different stages of regeneration, from wound healing to redevelopment (30–42 dpa). An integrated regulatory network was constructed by combining predicted miRNA target data with differential expression data (of miRNAs, mRNAs and proteins) to identify differentially expressed miRNAs and their potential targets. Notably, data were collected at different stages across the regeneration period and indicated that miR-233 was upregulated (compared to uninjured controls) during wound healing (3 dpa) and blastema formation (7 and 14 dpa). mRNA and protein of a putative target of miR-233, *col9a3* (*collagen type IX alpha 3 chain*) were also shown to be downregulated across all three time points. Further experiments using a luciferase reporter plasmid containing the *col9a3* 3′UTR showed that artificial miR-223 upregulation (using a miRNA mimic) led to a decrease in *col9a3. Col9a3* downregulation might facilitate actin cytoskeletal reorganization, a process implicated in cell migration during blastema formation.

Similarly, miR-133a was identified in the regenerating limb but was downregulated (compared to uninjured controls) during blastema formation (14 dpa) and early redevelopment (30 dpa). Experimentally induced downregulation of miR-133a led to an increase in the expression of its target *g6pd* (*glucose 6 phosphate dehydrogenase*, which encodes an enzyme involved in the pentose phosphate pathway). Both *g6pd* mRNA and G6PD protein were upregulated at 14 dpa and although there was an increase in the gene expression at 30 dpa, only the increase in G6PD protein was different from controls. This interaction might facilitate alternative metabolism in proliferating blastema cells, permitting rapid nucleotide synthesis [68]. Together, these studies emphasize the importance of performing integrative analysis of the microRNAome, transcriptome and proteome to identify candidate miRNAs, together with a more targeted approach to interrogate their role in a cell-type and stage and species-specific manner.

## 4. Spinal Cord Regeneration

A traumatic spinal cord injury has devastating consequences in mammalian species, often resulting in paralysis and loss of sensory function. The mammalian response to spinal cord trauma is characterized by a widespread apoptotic event surrounding the injury site, resulting in the death of neurons and glial cells. This is followed by the recruitment of many types of glial cells (including astrocytes, NG2^+^ (neuron-glial 2) glial and microglia), which form a glial scar around the injury site [69]. This glial scar releases inhibitory factors and also acts as a physical barrier preventing axon growth across the injury site, thus preventing regenerative repair [69]. Unlike mammals, regeneration-competent vertebrates do not form glial scar tissue and instead generate a permissive environment that promotes spinal cord repair [12,70,71,72,73].

**Figure 4:**
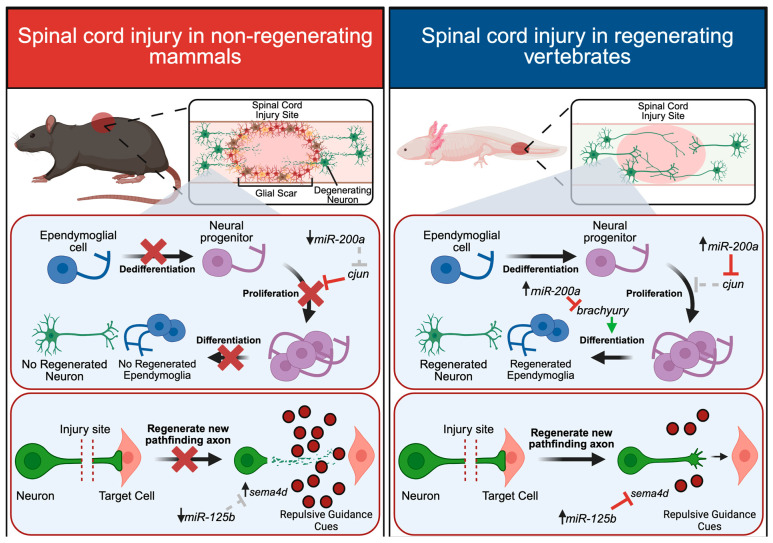
Cell-type responses to a spinal cord injury. A spinal cord injury in mammals (e.g rat) results in the formation of a physical barrier of glial cells around the injury site, known as the glial scar, and neurons surrounding the injury site rapidly degenerate. Ependymal glial cells do not dedifferentiate or proliferate and injured neurons do not regenerate pathfinding axons. miR-200a is downregulated, leading to the upregulation of its mRNA target *c-jun*, which prevents ependymoglia proliferation and promotes glial scar formation. miR-125b is also downregulated, leading to the upregulation of the repulsive guidance cue, *Sema4D* (*semaphorin 4D*) which further prevents any surviving neurons from extending new axons towards their synaptic targets. In regeneration-competent vertebrates (e.g., axolotl), no glial scar tissue forms, and neurons are able to extend new axons to re-establish lost connections with target cells. Ependymoglial cells dedifferentiate into neural progenitor cells which rapidly proliferate to ultimately differentiate into newly regenerated neurons or glial cells. miR-200a is upregulated to downregulate its target genes, *c-jun* and *brachyury*, which promote proliferation and differentiation into neurons or glia. Injured neurons are able to regenerate new pathfinding axons, which use external guidance cues to navigate towards their synaptic targets. miR-125b is upregulated after injury to downregulate the expression of the repulsive guidance cue, *Sema4D*. Created with BioRender.com.

While regenerative and non-regenerative animals exhibit stark differences in glial scar formation, they contain a similar cell-type architecture in the spinal cord, largely comprising ependymoglia and neurons. After a spinal cord injury in regeneration-competent vertebrates, ependymoglia surrounding the injury site rapidly proliferate, while in non-regenerating systems, they remain quiescent after injury [73]. This increase in ependymoglia proliferation is a critical driver of successful regenerative repair, with ependymoglial cells acting as stem cells that eventually differentiate into the necessary cell types required for regeneration. It is well documented that ependymoglial cells give rise to new neurons and glial cells to regenerate missing portions of the spinal cord in many regeneration-competent animals, including *Xenopus* [74,75], zebrafish [76], newts [77] and axolotls [78]. In some instances, ependymoglial cells can also give rise to new muscle and cartilage during tail regeneration [13,78], though many animals rely on the recruitment of Pax7^+^ (paired box) satellite cells to regenerate new tail muscle [74,79]. Recent fate-mapping studies have also revealed an additional layer of complexity to axolotl tail regeneration, showing that satellite cells not only give rise to newly regenerated muscle, but also maintain the ability to give rise to connective tissues [80]. Taken together, spinal cord and tail regeneration is driven by proliferating ependymoglia and satellite cells in the regeneration blastema that give rise to newly regenerated tissue (Figure 4).

### 4.1. microRNAs in Spinal Cord Regeneration

In the axolotl, miR-200a plays multiple roles in regulating both spinal cord and tail regeneration [12,13] (the mature strand is unknown in axolotl, though the human miRNA-200a is 3p). After a spinal cord ablation (in which a small portion of the spinal cord is removed rather than the entire tail), miR-200a is dramatically upregulated in ependymoglia within the spinal cord at 3 days post-injury. This upregulation of miR-200a is important for regulating the expression of its target gene, *c-jun*, which regulates ependymoglia proliferation [12]. In mammals, ependymoglia upregulate a protein complex comprising cFos and cJun, whereas the axolotl upregulates a non-canonical complex comprising cFos and JunB. This slight alteration of different Jun proteins is important for determining whether the protein complex will promote glial scar formation, or instead, promote ependymoglia proliferation without the formation of scar tissue. By targeting *c-jun*, miR-200a prevents the formation of the classic mammalian cFos/cJun complex, and JunB is instead recruited to the complex to ultimately promote functional regeneration in the axolotl [12] (Figure 4).

In another comparative study in the axolotl, the role of miR-200a was examined after both a spinal cord ablation and a tail amputation and was shown to play an important role in mediating the cell fate decisions of ependymoglial cells [13]. After a spinal cord ablation injury, miR-200a directly targets the mesoderm marker, *brachyury*, in ependymoglial cells [13]. This is particularly important for ensuring that ependymoglial cells differentiate into new neurons and glial cells rather than muscle (Figure 4). After a tail amputation, where there is more extensive tissue damage and muscle also needs to be regenerated, miR-200a was instead downregulated, leading to an upregulation in *brachyury* expression to promote ependymoglia differentiation into both mesoderm and ectoderm [13]. This study highlights a dual role for miR-200a in regulating cell fate decisions depending on different injury contexts, showing that different expression levels of miR-200a can determine the fate of stem cells during regenerative repair.

### 4.2. Neurite Outgrowth and Axon Guidance

While the necessity of ependymoglia is well-established in spinal cord repair, the regeneration of neurons and axons is also critical to establish functional recovery. Specifically, surviving or newborn neurons must regenerate pathfinding axons that navigate through their environment with extreme precision to re-establish lost synaptic connections. Importantly, various miRNAs have been shown to regulate both the extent of axonal regeneration and their pathfinding capabilities in a number of regeneration-competent animals [9,81,82]. For example, the use of transgenic zebrafish to overexpress pri-mir-9-5p resulted in a dramatic increase in the length of regenerating Mauthner axons in the spinal cord, indicating a prominent role for miR-9-5p in promoting neuronal regeneration. Furthermore, inhibiting the miR-9-5p target, *her6* (*hairy-related 6*) enhanced axon regeneration by modulating intracellular calcium activity [82].

In work using invertebrates, miRNAs have also been shown to be important for regulating axon regeneration in motorneurons. In regenerating snail motorneurons, miR-124-3p was shown to modulate the expression of *rock* (*rho-associated coiled-coil containing protein kinase*) [81], which has been shown to play an important role in actin disassembly and neurite extension during axon pathfinding [83,84,85]. The overexpression of miR-124-3p (by injection of a miR-124 mimic) abolished normal axonal responses to specific attractive guidance cues, but not to others [81], indicating cue-dependent functions during axonal pathfinding. Collectively, this work indicates that miRNAs can mediate neurite outgrowth and fast-acting neuronal responses to guidance cues which are required for functional regenerative repair.

In addition to modulating the extent or direction of regenerative neurite outgrowth, miRNAs can also regulate the expression of guidance cues that determine the direction of axon regeneration in the spinal cord. Studies in the axolotl revealed an important role for miR-125b (mature strand not specified in axolotl, but human miR-125b is 5p) in regulating the expression of *Sema4D* (*semaphorin 4D*) a repulsive guidance cue [9]. In the axolotl, *Sema4D* is expressed in cells lining the spinal cord, ultimately ensuring that axons remain appropriately constrained within the spinal cord during regenerative repair. When miR-125b expression was inhibited after injury (by injection of a miR-125b inhibitor), this resulted in an expansion in *Sema4D* expression within cells in the spinal cord itself, leading to aberrant axonal projections that extended outside of the spinal cord [9]. Increased levels of *Sema4D* thus impair the architecture of the spinal cord and likely prevent regenerating axons from reaching their proper synaptic targets. In exploring the role of miR-125b in a non-regenerating animal model, researchers discovered that miR-125b expression was dramatically reduced after a spinal cord injury in the rat. This resulted in high levels of expression of the repulsive guidance cue, *Sema4D*, ultimately contributing to the inhibitory environment of the glial scar that prevents axon growth in mammals. After injecting a miR-125b mimic into the injured rat spinal cord to overexpress miR-125b, a reduction in both *Sema4D* expression and the formation of glial scar tissue occurred. Importantly, rats injected with the miR-125b mimic exhibited significant improvements in locomotive abilities compared to control animals [9]. Taken together, these studies highlight the importance of miRNAs in regulating the regeneration of motorneurons and neurons within the spinal cord and demonstrate the potential therapeutic benefits of conserved miRNAs in regenerative repair.

## 5. Conclusions and Perspectives

While many reports from cell culture have enhanced our understanding of miRNAs in cellular repair, studying miRNAs in vivo is critical to encompass the complex environmental landscape that underlies tissue regeneration. Specifically, in vivo research of heart, limb and spinal cord regeneration in various animal models has been instrumental in identifying the role of miRNAs (and their potential gene targets) in tissue repair (Table 1). Some miRNAs, such as miR-99-5p/100-5p [8] and miR-125b [9], can target the same mRNA transcripts in regeneration-competent and -incompetent animals, and it has been shown that mere alterations in their expression patterns can drive a pro-regenerative response. Additionally, other studies have identified unique miRNAs that play important roles in mediating pro-regenerative behaviours such as cellular dedifferentiation or proliferation, but their roles in a non-regenerating animal model have not yet been explored.

Interestingly, different miRNAs can target the same signaling pathways through distinct mRNAs, indicating that altering the expression of proteins in the same signaling pathway may be beneficial in the repair of multiple tissues. For example, in zebrafish heart regeneration, miR-101a-3p regulates the expression of *fos* to regulate cardiomyocyte proliferation [10]. In axolotl spinal cord regeneration, miR-200a regulates *c-jun*, an important heterodimer binding partner of *fos*, to mediate ependymoglia proliferation [12]. Collectively, these studies highlight how different miRNAs are present in regeneration-competent species to regulate various components of the Fos signaling pathway to promote the proliferation of progenitor cells. It will be important in future studies to further explore how the same signaling pathways regulate the regeneration of different tissues and organs.

Interestingly, epimorphic regeneration shares certain features with carcinogenic tumors (i.e., cell division, migration, invasion and extracellular matrix remodeling) [67,89,90]. As such, some of the key miRNAs discussed in this review, such as miR-21-5p, miR-128-5p, miR-10b and miR-200, along with several others (miR-19b-2, miR-127-5p, miR-150-5p, miR-194-5p and miR-210-5p) have been characterized as prognostic markers of cancer. For example, miR-10b (discussed here in the context of axolotl limb regeneration) is upregulated in several types of primary cancer cells, targeting genes involved in invasion, migration and metastasis [91]. miR-21-5p, abundant during axolotl limb regeneration, also serves as a marker for cancer and targets the tumor suppressor gene *pdcd4* (*programmed cell death 4*) [92]. It should be noted, however, that although there might be molecular similarities between regeneration and cancer, regeneration is an incredibly organized process, whereas the development and progression of cancer is disordered [67]. The pleiotropic function of miRNAs makes them suitable candidates for targeting cancer cells, as the development of cancer arises from the accumulation of numerous genetic and epigenetic alterations, rather than a single mutation. Thus, tools developed for laboratory research and used to study animal regeneration (including miRNA mimics and antisense miRNA inhibitors) are now being tested in clinical trials as promising drug candidates for cancer treatment [91,93,94].

For any future potential for therapeutic targeting of miRNAs to promote mammalian regeneration, it will be important to continue comparative studies between regenerating and non-regenerating animals, to not only identify key miRNAs but also to compare their mRNA targets and signaling pathways during regenerative repair. As innovative techniques such as single cell sequencing become more accessible, this new technology will be important for identifying the specific signaling pathways present in different cell types that are up- or down-regulated in response to injury.

In future studies, it will not only be important to identify specific miRNAs that regulate tissue repair but to also explore how the expression of these miRNAs is regulated between species. To date, very few studies have uncovered the upstream regulators of miRNA activity, and it is somewhat unclear whether the expression of specific miRNAs is regulated at the genetic level [95,96] or post-transcriptionally [97,98]. In humans, multiple transcription factors such as c-Myb (MYB proto-oncogene), NF-Y (nuclear transcription factor Y subunit), Sp-1 (specificity protein 1), MTF-1 (metal regulatory transcription factor 1) and AP-2α (adaptor protein complex 2) are predicted to act as master regulators of miRNA expression [95]. However, the role of these transcription factors has not yet been explored in other species or in a regenerative context. While many transcription factors may in turn regulate miRNA gene expression, mature miRNAs have also been shown to regulate mRNA translation in a fast-acting manner, for example, by controlling local protein synthesis during axon guidance [99,100]. If miRNAs are able to act in such a time-sensitive manner, it is possible that their expression is not regulated at the genetic level, but rather post-transcriptionally. More recently, long non-coding RNAs have been shown to regulate miRNA activity by either directly binding to specific miRNAs to prevent their binding to target mRNAs, or by competing with miRNAs for mRNA binding sites [97,98]. In future, it will be important to explore all possible upstream regulators of miRNA activity and to examine whether these regulators differ between regenerating and non-regenerating animals. As we continue to examine the signaling pathways and cues that are required to elicit a regenerative response, miRNAs are exciting candidates that can be explored to understand the expression of pro-regenerative genes.

## Figures and Tables

**Table 1:** MicroRNAs regulating regenerative repair in animal models. List of mature miRNAs shown to play a role in regenerative repair using animal models. “Conserved target in mammals” column indicates whether the miRNA has been shown to target similar mRNA sequences in both regeneration-competent animals and mammals. Predicted targets were taken directly from the primary reference listed (if available); otherwise, the sequence provided was inputted into miRDB and the top 3 ranked human target genes are listed. Abbreviations: *smarca5* (*SWI/SNF-related matrix associated actin-dependent regulator of chromatin subfamily a*), *fntb* (*beta subunit of farnesyl-transferase*), *epdr1* (*ependymin related protein 1*), *znf197* (*zinc finger protein 197*), *thap2* (*THAP domain containing apopotosis associated protein 2*), *fosab* (*FBJ murine osteosarcoma viral oncogene homolog Ab*), *cpeb1a* (*cytoplasmic polyadenylation element binding protein 1a*), *stmn1a* (*survival motorneuron 1 gene*), *mkp1* (*mitogen-activated protein kinase phosphatase-1*), *islet1* (*ISL LIM homeobox 1*), *aff4* (*ALF transcription elongation factor 4*), *szrd1* (*SUZ RNA binding domain containing 1*), *kdm7a* (*lysine demethylase 7A*), *rpS29* (*ribosomal protein S29*), *rpL4/30* (*ribosomal protein L4/L30*), *mknk2 (mitogen-activated protein kinase-interacting serine/threonine-protein kinase*), *cadm2* (*cell adhesion molecule 2*), *tfap2c* (*transcription factor AP-2 gamma*), *Rhou* (*Ras homolog family member U*), *Gpd2* (*Glycerol-3-phosphate dehydrogenase 2*), *prdm11* (*PR/SET domain 11*), *rarb2* (*retinoic acid receptor beta 2*), *mmd* (*monocyte to macrophage differentiation associated*), *slc44a1* (*solute carrier family 44 member 1*), *hacd3* (*3-hydroxyacyl-CoA dehydratase 3*), *ptbp1* (*polypyrimidine tract binding protein* *1*), *her6* (*hairy-related 6*), *maml1* (*mastermind like transcriptional coactivator 1*), *bicc1* (*BicC family RNA binding protein 1*), *cdk8* (*cyclin dependent kinase 8*), *arrdc3* (*arrestin domain containing 3*), *vps13c* (*vacuolar protein sorting 13 homolog C*), *rock* (*rho-associated coiled-coil containing protein kinase*), *pxdnl* (*peroxidasin like*), *fbxo36* (*F-box protein 36*), *nes* (*nestin*), *Sema4D* (*semaphorin 4D*), *adamts4* (*ADAM metallopeptidase with thrombospondin type 1 motif 4*), *atoh8* (*atonal bHLH transcription factor 8*), *azi2* (*5-azacytidine induced 2*), *gfap* (*glial fibrillary acidic protein*), *cspg4/5* (*chondroitin sulfate proteoglycan 4/5*), *pax7* (*paired-box 7*), *bmp4* (*bone morphogenetic protein 4*), *zmynd11* (*zinc finger MYND-type containing 11*), *nr6a1* (*nuclear receptor subfamily 6 group A member 1*), *slc9a6* (*solute carrier family 9 member A6*).

**Heart** **Regeneration**						
**miRNA**	**Role**	**Target(s)**	**Animal**	**Reference**	**Conserved target in mammals**	**Predicted Target(s)**
miR-99-5p/100-5p	Cardiomyocyte proliferation and dedifferentiation	*Smarca5* *fntb*	Zebrafish and Mice	[8]	Yes	*epdr1* *znf197* *thap2*
miR-101a-3p	Cardiomyocyte proliferation Prevents scar formation	*fosab (fos)*	Zebrafish	[10]	Unknown	*cpeb1a* *stmn1a* *mkp1*
miR-128-3p	Deposition of extracellular matrix Non-myocyte hyperplasia	*islet1*	Red Spotted Newt	[86]	Unknown	*aff4* *szrd1* *kdm7a*
**Limb** **Regeneration**						
**miRNA**	**Role**	**Target**	**Animal**	**Reference**	**Conserved target in mammals**	**Predicted Target(s)**
miR-10b-5p	Protein synthesis	*rpS29* *rpL30* *rpL4*	*Pleurodeles waltl* and *Notophthalmus viridescens*	[17]	Unknown	*mkn2* *cadm2* *tfap2c*
miR-21-5p		*jagged1*	Axolotl	[66]	Unknown	*Rhou* *Gpd2* *prdm11*
**Spinal Cord &** **Motorneuron Regeneration**						
**miRNA**	**Role**	**Target**	**Animal**	**Reference**	**Conserved target in mammals**	**Predicted Target(s)**
miR-1-3p	Tail growth	*rarb2*	*Notophthalmus viridescens*	[87]	Unknown	*mmd* *slc44a1* *hacd3*
miR-133a-3p	Tail growth	*rarb2*	*Notophthalmus viridescens*	[88]	Unknown	*ptbp1* *maml1* *bicc1*
miR-9	Axon growth	*her6*	Zebrafish	[82]	Unknown	*cdk8* *arrdc3* *vps13c*
miR-124-3p	Axon pathfinding	*rock*	*Lymnaea* *stagnalis*	[81]	Unknown	*pxdnl* *fbxo36* *nes*
miR-125b	Axon growth	*Sema4D*	Axolotl and Rat, Zebrafish	[9]	Yes	*adamts4* *atoh8* *azi2*
miR-200a	Axon growth Glial scar formation Cell proliferation	*c-jun*	Axolotl	[12]	Unknown	*gfap* *vimentin* *cspg4/5*
	Stem cell fate decisions	*brachyury*	Axolotl	[13]	Unknown	
miR-196-3p	Cell proliferation	*pax7* *bmp4*	Axolotl	[15]	Unknown	*zmynd11* *nr6a1* *slc9a6*

## Data Availability

No new data were created or analyzed in this study. Data sharing is not applicable to this article.
